# Natural tannins as anti-SARS-CoV-2 compounds

**DOI:** 10.7150/ijbs.74676

**Published:** 2022-07-11

**Authors:** Shao-Chun Wang, I-Wen Chou, Mien-Chie Hung

**Affiliations:** 1 Graduate Institute of Biomedical Sciences, College of Medicine, China Medical University, Taichung, Taiwan, 40402; 2 Center for Molecular Medicine, China Medical University Hospital, Taichung, Taiwan, 40447; 3 Research Center for Cancer Biology, China Medical University, Taichung, Taiwan, 40402; 4 Department of Biotechnology, Asia University, Taichung, Taiwan, 41354

## Abstract

Tannins are polyphenols enriched in wood, bark, roots, leaves, seeds and fruits of a variety of plants. Over the last two decades, there has been an increasing interest in understanding the biological functions of tannins and their applications as antioxidants, anticancer drugs, and food additives. Since the outbreak of the COVID-19 pandemic, much effort has been devoted to finding an expedient cure. Tannins have been put forward as having possible anti-COVID-19 properties; however, owing to the profuse nature of the structurally diverse derivatives of tannins, the tannin species in the family associated with an indication of anti-COVID-19 have been poorly defined, compounded by frequent terminology misnomers. This article reviews the tannin family in fruits and the current knowledge about the activities of the compounds with regard to severe acute respiratory syndrome coronavirus 2 (SARS-CoV-2). It will aid molecular and cellular biologists in developing natural anti-viral chemicals as means of overcoming the current and future pandemics.

## Introduction

Beginning in early 2020, at time of writing the COVID-19 coronavirus pandemic has lasted for over two years, with about 507 million cases of infection and more than 6.2 million deaths (April 2022; Global Change Data Lab). Several anti-COVID vaccines have been rushed into the clinic globally, debuting mRNA technology in vaccines. However, although vaccination has significantly reduced the extent and duration of symptoms, the efficacy of anti-viral immunity declines rapidly and breakthrough infections frequently occur even in the fully vaccinated population 1-3. Thus, on the edge of surging infections worldwide, coexisting with the virus is becoming a more realistic solution 4, 5. Thus, in the post-pandemic world, regimens including anti-SARS-CoV-2 small molecules are highly desirable. Currently, three SARS-CoV-2 inhibitors, remdesivir/VEKLURY (Gilead) 6, ritonavir/PAXLOVID (Pfizer) 7, and molnupiravir/LAGEVRIO (Merck) 8 have been authorized by the US FDA to treat patients with symptomatic SARS-CoV-2. However, they are mostly used for treatment of patients with mild-to-moderate symptoms 1, 9. Furthermore, concerns about the effectiveness of these drugs in blocking the emerging variants and the potential mutagenic activity of molnupiravir in causing viral variants have been raised 10, 11. Alternatively, repurposing the existing FAD-approved drugs may also be a promising strategy for timely development of anti-COVID molecules. For example, multiple groups have reported that imatinib/GLEEVEC, a clinically approved targeted tyrosine kinase inhibitor in cancer therapy, is effective in suppressing COVID-19 infection through different mechanisms 12-14.

Considering the fact that living with infectious pathogens such as coronaviruses is likely to be a norm in the future world 15-17, safe and potent anti-viral compounds with the potential to become acute treatments as well as chronic prevention methods are becoming an attractive approach 18. Intuitively, natural products are the promising sources of such chemicals 19, 20. A long track record of past and recent studies has demonstrated the activities of the abundant tannins derived from numerous common fruits in suppressing viral infection (see below). This review summarizes the current understanding of the tannin compounds which are relatively abundant in fruits, for their biological activities, with particular focus on COVID-19 inhibition. Reviews of the chemistry and biochemistry of the naturally occurring tannins compounds can be found elsewhere by others 21-23.

## Classification of tannins

Characterization based on chemical structures classifies tannins into different categories: hydrolysable tannins, condensed tannins, complex tannins, and phlorotannins. Phlorotannins, are of low abundance and present mainly in brown macroalgae; however, tannins of the other categories are abundant in terrestrial plants 24. Hydrolysable tannins can be further subdivided into gallotannins, commonly referred to as tannic acid (TA) and ellagitannins such as punicalagin. Gallotannins are relatively rare in nature 25. The complex tannins are structurally characterized by a gallotannin or ellagitannin unit followed by a catechin unit. Condensed tannins include proanthocyanidins, catechins, and procyanidins, and are the most abundant of all tannins followed by the hydrolysable tannins 26. The hydrolysable tannins are prone to oxidation 27 and characterized by their short half-lives 28. In an oxidative environment, tannic acid can be rapidly degraded into gallic acid 29. Due to the unstable nature of tannic acid, there have been only few studies investigating its exact content in natural products or wines. In some other studies, the nomenclatures are overlap in tannic acid and other tannins.

## Levels of tannins in wine and fruits

Some of the winemaking factors determining the flavor of wine products are the phenolic substances extracted from the seeds, skin, and stems of grapes as well as the wooden casks during the fermentation process. Due to technical limitations and short half-lives, the exact contents of tannic acid in different wines or fruits have been poorly characterized. Most studies measured the tannin content rather than tannic acid. It was indicated in some studies that hydrolysable tannins do not exist naturally in grape-derived wines 30. Others identified tannic acid in grape-derived wines sourced from the wooden barrels and the various parts of grapes during the temperature-dependent brewing process 31, 32. Depending on the winery, tannic acid can be added to the wines to enhance the astringent taste 33. Tannic acid in the solution can interact with the major wine protein VVTL1 (*Vitis vinifera* Thaumatin-Like-1), resulting in conformational change of the protein VVTL1 as demonstrated by circular dichroism spectra in the near-UV region 30. It is estimated that the tannic acid content (dry weight) is 4.1g/100g in grape seeds 34. For tannins in general, their contents are relatively high in fruits such as persimmon, pomegranate, grape, cashew apple, guava, chokeberries, blackberries, raspberries, apple, banana, etc 25, 26, 35, 36. The sources and analytical methods of studies measuring tannins are summarized in **Table 1**. Most grape-derived tannins are condensed tannins 37. In grapes and berries, it has been estimated that 80% and 20% of tannins are from seed and skin, respectively 38. In wines, 60% of total tannins are generated from grape skin 38. The average tannin concentration of over 1300 commercial red wines from different regions (California, Oregon, Washington, Australia, and France) was 544 ± 293 mg/L catechin equivalents (CE). Among them, the top five high-tannin red wines are Cabernet Sauvignon (672 mg/L CE), Zinfandel (652 mg/L CE), Merlot (559 mg/L CE), Syrah (455 mg/L CE) and Pinot noir (348 mg/L CE) 39. Thus, tannin concentrations in wines vary dramatically.

## The biological effects of fruit-derived tannins

The health benefit of red wine extracts as nutritional supplements is a popular topic on social media. However, little research provides controlled experimental evidence about the effects of red wine extracts on human subjects. The biological effects of tannins in natural products and wines have been investigated by different groups (**Table 2**). Tsuchiya *et al.*, for example, showed that administration of a test beverage including 200 mg/day oligomeric procyanidins (condensed tannins) isolated from red wines improved skin condition of moisturizing and whitening in a group of 30-60 year-old healthy women in 12 weeks 40. Kitada *et al.* reported that daily supplementation of resveratrol (19.2 mg) and polyphenols (136 mg) extracted from red wine for 8 weeks improved the metabolic profile including enhancing insulin sensitivity and decreasing serum low-density lipoprotein-cholesterol as well as triglyceride in non-diabetic individuals 41. Moreover, Ueda *et al.* demonstrated the anti-viral effects of persimmon-derived tannins against viruses including influenza virus, herpes simplex virus-1 and vesicular stomatitis virus by inhibition of viral infection 42. Mechanistically, it has been shown that the catechol units of tannic acid function as scavengers of reactive oxygen species (ROS), contributing to the anti-oxidative, anti-inflammatory and anti-carcinogenic activities of tannic acid 43, 44.

Dietary supplementation with tannic acid helped improve cognition and behavioral dysfunctions, in part by inhibiting the processing of amyloidogenic precursor proteins, reducing the production and cerebral vascular deposits of β-amyloid, and reducing neuroinflammation manifested by β-amyloid plaque-associated microgliosis in aged Alzheimer-like transgenic mice 45. Tannic acid protected rat kidneys after ischemia-reperfusion from oxidative stress through activation of nuclear factor erythroid-2-Related factor 2 (NRF2) 46. Subcutaneously administered tannic acid decreased myocardial fibrosis and reduced the levels of inflammatory cytokines and apoptosis-associated mediators such as toll-like receptor 4 (TLR4), Nuclear factor kappa B (NF-κB), B-cell lymphoma-2 (Bcl-2), Bcl-2-associated protein (Bax) and the stress-responding kinase p38 in a myocardial fibrosis mouse model induced by isoproterenol 47. Additionally, tannic acid reduced kidney damage modulated by NF-κB and NRF2 pathways, resulting in the reduction of oxidative stress, apoptosis and inflammatory cytokines such as IL-6, IL-8 and TNF-α in an arsenic trioxide-induced nephrotoxicity rat model 48.

The anti-inflammatory and anti-oxidant activities of tannic acid were exploited by a nanogel-based delivery system into the murine macrophage cell line RAW264.7. Treatment with tannic acid significantly lowered the levels of intracellular ROS, TNF-α and IL-6 induced by phorbol 12-myristate 13-acetate (PMA) 49 as an inflammation activator. Similar benefits have also been observed in a mouse model of zymosan-induced peritonitis 50. In a chronic alcohol-induced mice model, treatment with tannic acid-encapsulated Poly D, L-lactide-co-glycolic acid (PLGA) nanoparticles mitigated the levels of triglyceride, total cholesterol, and the inflammation cytokines such as TGF-β, IL-6, TNF-α and IL-1β, resulting in lowering liver oxidative stresses, hepatic apoptosis, and promoting functional recovery 51. It was proposed that the hepato-protection of tannic acid is through its binding with the receptor tyrosine kinase receptor EGFR, which in turn activates the EGFR-AKT-STAT3 pathway 51.

The potential anti-tumor activities of tannins have been explored in numerous studies. The addition of tannic acid in cholangiocarcinoma chemotherapeutic regimens such as mitomycin C and 5-fluorouracil can achieve a synergistic effect which is believed to result from modulating the drug efflux pathways by tannic acid, thus increasing chemotherapy-induced apoptosis by the treatment 52.

It seems that the function of tannic acid in mediating oxidoreductive stresses is cell context dependent. In contrast to its anti-ROS anti-inflammatory cell-protective activities as observed in non-transformed cells, tannic acid induced apoptosis in prostate cancer cells through increasing the level of ROS, inducing ER stress, inhibiting tumor lipogenesis, and disrupting the plasma membrane as well as the nuclear structure 53. It is also reported that tannic acid possesses a dual pathway to induce human embryonic carcinoma apoptosis 54. Tannic acid repressed the Wnt/β-catenin signaling pathway and induced TNF-related apoptosis-inducing ligand (TRAIL)-mediated apoptosis by increasing ROS levels in the mitochondria 54. Fruits and vegetable juices enriched with biologically active phytochemicals have been suggested to contribute to proliferation inhibition and apoptosis in breast cancer cell lines such as MCF-7 and MDA-MB-231, as well as suppression of breast cancer xenograft tumor growth in rodent models 55. Indeed, fruit and juice consumption is associated with reduced risk of breast cancer in a dose-dependent manner in a cohort study 56. It is speculated that the polyphenols of the fruits and vegetables are the main source of the anti-inflammatory and anti-oxidant components which in turn inhibit cancer development 55.

## The anti-COVID-19 activities of tannins

The two major frontiers in battling the COVID-19 are infection and propagation of the SARS-CoV-2 coronaviruses, and the possible extreme reaction of the body's immune system in response to pathogenic intrusion 57. While most research efforts have been devoted to preventing viral infection or eradicating the virus from the body, relatively less attention has been focused on developing methods to cope with the inflammatory responses, the “cytokine storm”, over the course of COVID-19. Elevations of IL-6, IL-1β, TNF-α, IFN-γ and IL-10, as well as reductions in CD4^+^ and CD8^+^ T lymphocytes have been observed in COVID-19 patients, especially in severe cases 58. Currently, several polyphenol-based 59 and tannin-based clinical trials (NCT04911777 60, NCT04403646 61, and IRCT20200418047122N1 62) have been conducted with COVID-19 patients. Oral tannins extracted from quebracho and chestnut in combination with B12 vitamin and standard treatment reduced macrophage inflammatory protein-1α (MIP-1α) and TNF-α levels in hospitalized COVID-19 patients 63. In a Syrian hamster model of COVID-19, persimmon-derived tannins pre-administered by oral gavage were able to suppress SARS-CoV-2 titers, reduce the severity of pneumonia and decrease inflammation-related gene expression such as IL-6, TNF-α, and IFN-γ 64.

**Figure 1:**
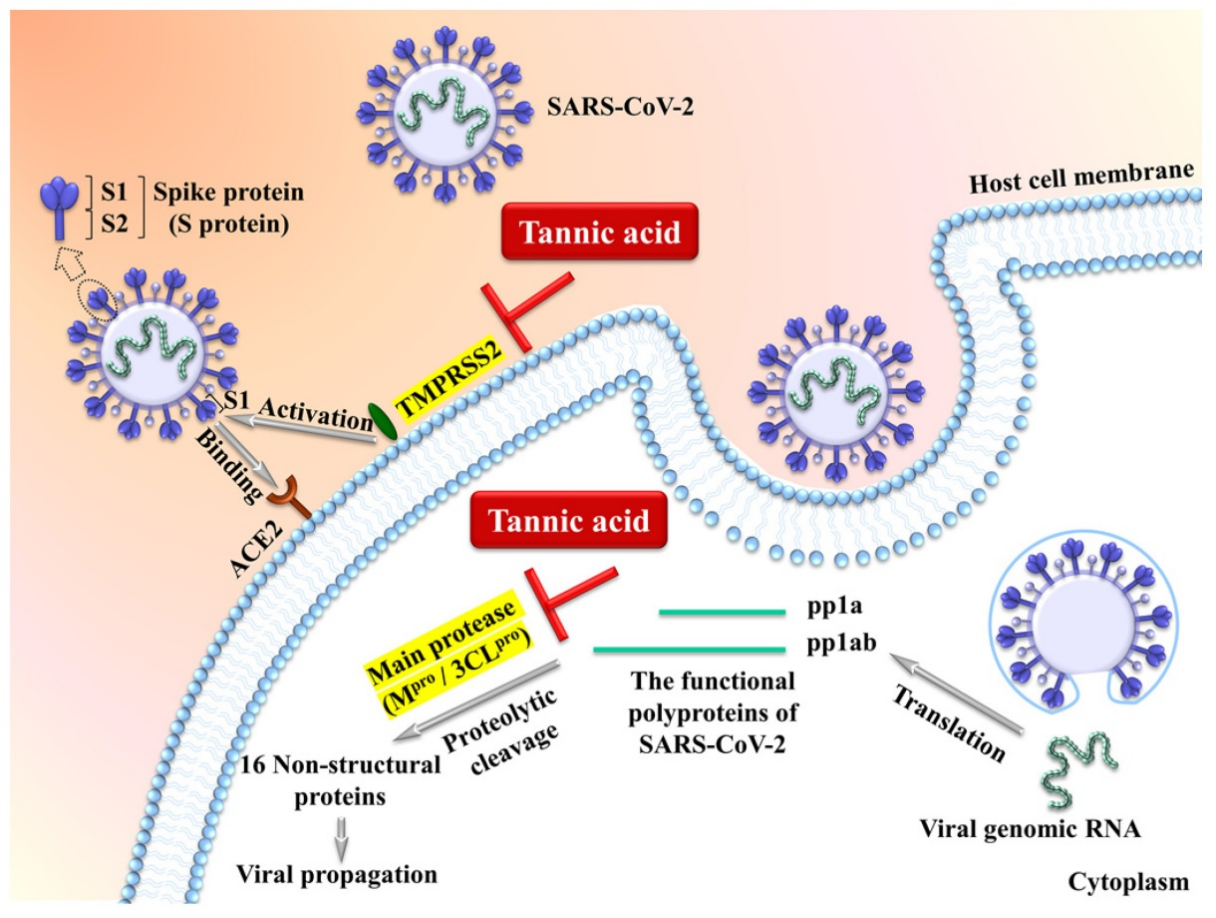
Tannic acid acts as a potent dual inhibitor of the cell surface protease TMPRSS2 and the viral main protease (M^pro^/3CL^pro^) to inhibit the SARS-CoV-2 activity 65-70.

The transmembrane protease serine 2 TMPRSS2 is an essential cell surface protease which processes the viral surface spike protein (S protein) to enhance its binding to the cell surface receptor ACE2 (angiotensin converting enzyme 2) followed by cell entry via membrane fusion. The intracellular viral genomic RNA is then translated into polyproteins which are subject to further proteolytic cleavage by the viral main protease/3-chymotrypsin-like cysteine protease (M^pro^/3CL^pro^) to produce non-structural functional proteins essential for viral propagation (**Figure 1**) 65-70. It has been demonstrated by multiple groups that tannic acid is a potent dual inhibitor of TMPRSS2 and M^pro^/3CL^pro^ to inhibit the SARS-CoV-2 activity 65-69. Tannic acid inhibits M^pro^/3CL^pro^ with IC_50_ ranging from 1 μM to 13.4 Μm 65-69, and inhibits TMPRSS2 with IC_50_ ranging from 2.31 μM to 50 μM 68, 69. The variable IC_50_ may result from the different methodologies such as fluorescence resonance energy transfer and ELISA-based enzymatic assays. Importantly, the dual inhibition of TMPRSS2 and M^pro^/3CL^pro^ by tannic acid can be translated into suppression of cellular entry of the virus 68. Thus, tannic acid shows high potential for inhibition of SARS-CoV-2 among plant-derived polyphenols. Similar inhibitory activities of M^pro^/3CL^pro^ by the tannins of dimeric proanthocyanidins 71, punicalagin 72 and mixtures of tannic acid with plant-derived polyphenols 67 have also been observed in *in vitro* studies.

Consistently, it has been reported that green tea-derived tannins inhibited viral replication of SARS-CoV-2 *in vitro* and stably persist in the pharyngeal mucosa 1 hour after administration by throat spray 73, supporting the potential of green tea tannins in anti-COVID-19 therapies. Moreover, the hydrolysable tannins such as pedunculagin, tercatain, and castalin have been demonstrated to be potential inhibitors of SARS-CoV-2 using the molecular operating environment software (MOE 09) which is an integrated computer-aided molecular design platform 74. These natural compounds are predicted to be able to bind to the catalytic dyad residues of M^pro^/3CL^pro^ and inhibit the enzyme activity 74. It is worth mentioning that oral administration of highly-purified isomers of tannic acid exerts anti-COVID-19 activity against both the omicron and delta variants 60. A phase III clinical trial will be carried out in 2022 (NCT04911777).

## Conclusion

Substantial evidence supports the ameliorating functions of tannins to treat diseases involving metabolic dysregulation and protect against cancer through opposing the damage caused by oxidative stresses and inflammatory insults. A large body of studies highlights the therapeutic potential of tannins, particularly tannic acid, in preventing and treating of SARS-CoV-2 infection. Interestingly, a high-efficiency particulate air (HEPA) filter coated with tannic acid has been developed which showed rapid and highly efficient capability to capture the influenza A virus, revealing a new application of tannic acid to constrain virus spread 75. Thus, it is expected that tannin derivatives and natural source products can be exploited to develop safe and promising strategies in responding to globally emerging new viruses or other pathogens.

As the world is evolving into the post-pandemic era of COVID-19 with potential unknown variants arising in the horizon, tapping the natural sources for safe and effective medication is an attractive resort. As summarized, a body of studies support that incorporating daily diet with the source food of anti-viral activity can have protective or ameliorating effects to curb viral infection. Thus, it is of high public interests to entail a clinically defined dieting practice for disease control. For example, the known concentrations of the different tannins required for viral inhibition can be used as a guidance to develop the formulae consisting of quality-defined natural products such as tea and fruits for daily consuming by the general population. Furthermore, given the potency of the tannin derivatives in curtailing SARS-CoV-2 infection *in vitro*, compounds derived from modified tannins can be exploited for the development of anti-COVID drugs. Finally, it is worth exploring to determine whether combination of the tannin-based drugs with the FDA-approved COVID-19 medicines can enhance the therapeutic efficacy. The progression in these directions may also be applied to other viruses and help us prepare to fight the arising health-threatening pathogens in the future.

## Figures and Tables

**Table 1:** The sources and analytical methods of tannins

Research title	Source	Tannin type	Method	Ref.
Comparison of analytical methods for the determination of condensed tannins in grape skin	38 grape skin samples	Condensed tannin	protein and methylcellulose precipitation and by HPLC-phloroglucinolysis	31
Inhibitory effect of tannic acid extracted from grape seeds and pomegranate peels on some microorganisms	Seeds of local grape fruit and peels of pomegranate fruit	Hydrolysable tannin (tannic acid)	Chromatographic separation and automated analysis	34
Tannin contents of selected plants used in Jordan	Anise, broad beans, chamomile chard, chickpea, clary, cocoa, coriander, dates, eggplant, fennel, fenugreek, figs, garden rocket, gundelia, hawthorn, Jew's mallow, lentils, liquorice locust, mint, oak, okra, parsley, plums, pomegranate, red and green grapes, rosemary, sage, sumac, tea, thyme, tomatoes, turmeric, verbena, vine leaves, wheat	Total tannins	HPLC	76
Antioxidant properties of different fruit seeds and peels	Gooseberry, watermelon, apple, plum, melon, red grapes, white grapes, lemon, red grapefruit, white grapefruit, kiwi fruit, orange	Total tannins	Folin-Ciocalteu method	36
Variability of tannin concentration in red wines	1325 commercial red wines	Total tannins	Protein precipitation	39
Tannins in fruit juices and their removal [REVIEW]	Fruit juice (apple, banana, cashew apple, guava, grape, jamun, persimmon, pomegranate, myrobalan, sugarcane)	Total tannins; condensed tannins, hydrolysable tannins (punicalagin, ellagic acid)	protein precipitation method, Folin-Denis method	35
Technological application of tannin-based extracts [REVIEW]	Nut, black currant, strawberry, European blackberry, black raspberry, Pomegranate, Guava, mango, almond.	Condensed tannins, hydrolysable tannins	HPLC, vanillin-HAL, gas chromatography, spectrophotometric techniques, radial diffusion, acid butanol (spectrophotometric) assays	26
Proanthocyanidins and hydrolysable tannins: occurrence, dietary intake and pharmacological effects [REVIEW]	Fruits (cranberries, chokeberries, plums, black diamond, blueberries, black currants, red currants, blackberries, crowberries, lingonberries, red grapes, grape seeds, strawberries, peaches, apricot, raspberries, pears, apple, pomegranate, guava, mango), juice (cranberry, apple, grape), nuts (almonds, hazelnuts, pecans, pistachio nuts, walnuts), legumes (beans, carob fiber, cowpeas, lentils, peanuts), cereal grains (barley, buckwheat, sorghum, rice), beverages (wine, tea), cacao beans, chocolate	Condensed tannin (proanthocyanidins), hydrolysable tannins (ellagitannins and gallotannins)	HPLC	25
Health effects, sources, utilization and safety of tannins: a critical review [REVIEW]	Fruits (majuphal, babul, amla, ripened banana, red supari, munakka, dates, raisins, badillayachi, persimmon, mango, samgiri), leafy vegetables (canola, drumstick, bathua, gotu kola, joseph's coat, edible amaranth, fenugreek, desert horse purslane, plumed cockscomb, snakeroots, punarnava, Mexican mint, false amaranth, benghal dayflower, shona cabbage, buttercup, white gulmohur, musk thistle), cereals and millets (rice, wheat, sorghum, bajra, ragi, finger millets, pearl millet), seeds/nuts (cumin seeds, mango seeds, fenugreek, coffee, castor seeds, faba beans, tamarind seeds, almond, brazil nut, cashew nut, virginia peanut, walnut, pistachio, pecan, pine nut, hazelnut, macadamia nut), legumes (pigeon pea, chickpea, green gram, red gram, soya bean, kidney bean, cowpea)	Total tannins	Spectrophotometric method, HPLC, Folin-Denis method	77

**Table 2:** Administration and effects of tannins in humans.

Tannin source	Type of tannin	Route	Disease (the number of subjects)	Biological effects	Outcome-based potential benefits for COVID-19 prevention and cure	Ref.
Grape seed extract	Condensed tannins (proanthocyanidins)	Oral	Healthy (8)	Lipid hydroperoxide↓; oxidants↓; antioxidant capacity↑; resistance to oxidative modification of LDL↑; prevent plasma postprandial oxidative stress in humans.	Antioxidant capacity↑	78
Grape seed extract	Total tannins	Oral	Metabolic syndrome (27)	Systolic and diastolic blood pressures↓; no significant changes in serum lipids or blood glucose values.	---	79
Lyophilized grape powder	Total tannins (grape polyphenols)	Oral	Women (24 premenopausal and 20 postmenopausal)	Triglyceride↓; LDL↓; apolipoproteins B and E↓; cholesterol ester transfer protein activity↓; whole-body oxidative stress↓; plasma TNF-α↓; exert a cardioprotective effect by lowering plasma lipids and reducing oxidative stress.	Plasma TNF-α↓	80
Flavonoids and phenolic acids from cranberry juice	Condensed tannins (proanthocyanidins)	Oral	Healthy postmenopausal women (10)	Plasma total antioxidant capacity↑; resistance of LDL against oxidation↑	Plasma total antioxidant capacity↑	81
Bath additive containing tannic acid	Hydrolysable tannin (tannic acid)	Bath	Atopic dermatitis (21)	Improvement of pruritus↑	---	82
A tannic acid-based medical food, Cesinex®	Hydrolysable tannin (tannic acid)	Oral	Diarrhea (10)	Diarrhea↓; transepithelial resistance↑; CFTR-dependent or the calcium-activated Cl^-^ secretion↓; improve the impaired epithelial barrier function induced by TNFα; Cesinex® has high antioxidant capacity.	Improve the impaired epithelial barrier function induced by TNFα; high antioxidant capacity	83
Grape seed proanthocya-nidin	Condensed tannins (oligomeric proanthocya-nidin)	Oral	Asymptomatic carotid plaques or abnormal plaque free carotid intima-media thickness (287)	progression of carotid atherosclerotic plaques↓; carotid plaque size↓; the number of plaques↓; incidence rate for transitory ischemic attack↓; arterial revascularization procedure↓; clinical vascular events↓	---	84
Cherry juice	Condensed tannins (procyanidin B2)	Oral	Insomnia (11)	sleep time↑; sleep efficiency↑; serum kynurenine to tryptophan ratio↓; serum PGE2↓; inflammation↓; inhibition of inhibited indoleamine 2, 3-dioxygenase with a reduction in the degradation of tryptophan	Serum kynurenine to tryptophan ratio↓; serum PGE2↓; inflammation↓; inhibition of inhibited indoleamine 2, 3-dioxygenase	85
Grape seed extract	Total tannins	Oral	Postmenopausal women (46)	Grape seed extract did not decrease plasma estrogens (estrone, estradiol, estrone sulfate) and did not increase precursors of androgens (testosterone and androstenedione).	---	86
Flavonoid-rich blueberry beverage	Condensed tannins (antho- and pro-cyanidins)	Oral	Healthy (18)	Improved cognitive function.	---	87
Flavonoid-rich apple with skin	Total tannins	Oral	Healthy (30)	Improves endothelial function assessed using flow-mediated dilation of the brachial artery in individuals at risk for cardiovascular disease.	---	88
Red grape seed extract	Total tannins	Oral	Mildly hyperlipidemia (52)	Total cholesterol↓; LDL cholesterol↓; Ox-LDL↓; the risk of atherosclerosis and cardiovascular disorders↓	---	89
Cranberry beverage	Total tannins	Oral	Healthy (54)	Human γδ-T cell proliferation (percentage of the CD3^+^ population)↑; the number of symptoms associated with colds and influenza↓; the ability of PBMC to secrete IFN-γ ↑	Human γδ-T cell proliferation (percentage of the CD3^+^ population)↑; the ability of PBMC to secrete IFN-γ ↑	90
Grape seed extract	Condensed tannin (proanthocyanidins)	Oral	Heathy (11)	Condensed tannin does not affect iron status or bioavailability.	---	91
Actitan F	Total tannins	Oral	Acute gastroenteritis (60)	The number of stools↓	---	92
Persimmon fruit tannin-rich fiber	Condensed tannins	Oral	Healthy (40)	Plasma total cholesterol levels↓; plasma low-density lipoprotein cholesterol levels↓	---	93
Oligomeric proanthocya-nidins of red wine	Condensed tannins	Oral	Healthy (100)	skin whitening and moisturizing↑	---	40
Red wine extract	Total tannins	Oral	Healthy (12)	Insulin sensitivity↑; peripheral blood mononuclear Sirt1 and p-AMPK↑; LDL-C↓; triglyceride↓; IL-6↓	Peripheral blood mononuclear Sirt1 and p-AMPK↑; IL-6↓	41
